# Disease Model Distortion in Association Studies

**DOI:** 10.1002/gepi.20576

**Published:** 2011-03-17

**Authors:** Damjan Vukcevic, Eliana Hechter, Chris Spencer, Peter Donnelly

**Affiliations:** 1 Wellcome Trust Centre for Human Genetics, University of OxfordOxford, United Kingdom; 2 Department of Statistics, University of OxfordOxford, United Kingdom

**Keywords:** linkage disequilibrium (LD), nonmultiplicative, nonadditive, interaction, epistasis, genome-wide association study (GWAS), case-control, tag SNP

## Abstract

Most findings from genome-wide association studies (GWAS) are consistent with a simple disease model at a single nucleotide polymorphism, in which each additional copy of the risk allele increases risk by the same multiplicative factor, in contrast to dominance or interaction effects. As others have noted, departures from this multiplicative model are difficult to detect. Here, we seek to quantify this both analytically and empirically. We show that imperfect linkage disequilibrium (LD) between causal and marker loci distorts disease models, with the power to detect such departures dropping off very quickly: decaying as a function of *r*^4^, where *r*^2^ is the usual correlation between the causal and marker loci, in contrast to the well-known result that power to detect a multiplicative effect decays as a function of *r*^2^. We perform a simulation study with empirical patterns of LD to assess how this disease model distortion is likely to impact GWAS results. Among loci where association is detected, we observe that there is reasonable power to detect substantial deviations from the multiplicative model, such as for dominant and recessive models. Thus, it is worth explicitly testing for such deviations routinely. *Genet. Epidemiol*. 35: 278-290, 2011.  © 2011 Wiley-Liss, Inc.

## INTRODUCTION

Genome-wide association studies (GWAS) exploit the correlation structure in the genome, due to linkage disequilibrium (LD), by testing a representative subset of genetic markers for association with disease. As a result, we expect GWAS to highlight markers *correlated* with causal loci rather than to discover the causal loci directly. Depending on the strength of LD between causal and marker single nucleotide polymorphisms (SNPs), the observed disease effect at the marker will be an imperfect representation of the true disease effect.

In this paper, we study a particular aspect of this relationship both analytically and empirically. We consider case-control studies that genotype SNPs and disease models with either a single SNP or a pair of interacting SNPs. We ask two related questions. First, how do the disease model parameters (“effects”) change as the LD between the causal and tag SNPs diminishes? Second, how does the power to detect departure from the multiplicative model change? We show that as the LD between causal and marker loci decreases, nonmultiplicative and interaction effects decay faster than multiplicative effects, quadratically rather than linearly. This makes the former harder to detect; stated in terms of power, the decay is quartic rather than quadratic. Furthermore, compared to the true disease model, the apparent disease effect as observed at marker SNPs will be distorted to look more like a multiplicative one.

The impact of imperfect LD has been well characterized for multiplicative models, both in terms of effect sizes and power [Chapman et al., 2003; Pritchard and Przeworski, 2001; Zondervan and Cardon, 2004]. Measuring the LD using the squared correlation (*r*^2^), a well-known rule of thumb is that a sample size of roughly *N/r*^2^ is required at a marker in order to have the same power to detect an association as a study with sample size *N* that types the causal SNP [Pritchard and Przeworski, 2001]. Here we derive a similar result, showing that a sample size of about *N/r*^4^ is required to maintain equivalent power to detect a deviation from a multiplicative model.

We derive an analogous result for a scenario involving two interacting SNPs under a simple interaction model. Specifically, suppose we type a marker SNP for each of the two causal SNPs, with the LD between each pair being 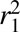 and 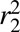, respectively. We show that a sample size of roughly 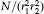 would then be required for equivalent power to detect the interaction as a test with sample size *N* that types the causal SNPs directly.

The above results apply for any given, fixed, marker loci. To study the impact of distortion on actual GWAS outcomes, we perform a simulation study with empirical patterns of LD. We find that loci highlighted by GWAS will often be highly correlated with the causal SNP, limiting the amount of distortion observed. When this is the case, there will be reasonable power to detect substantial departures from the multiplicative model, such as for recessive and dominant models. Therefore, there is value in testing for such departures routinely.

Previous studies have explored the impact of LD on GWAS. Most have done so empirically, and only for multiplicative models at single SNPs [e.g. Spencer et al., 2009]. At least two studies go further: Bhangale et al. [2008] considered recessive and dominant models empirically; Zheng et al. [2009] studied nonmultiplicative models analytically assuming the same allele frequency at the causal and marker SNP. Our study is more extensive: we characterize the effect of LD on power analytically, we do not impose restrictions on allele frequencies, and we study interactions as well as single-SNP models.

While we focus on case-control studies, we note that some related work has been published for studies of quantitative traits using variance components models. Sham et al. [2000] derived similar results for the impact of LD, and Hill et al. [2008] showed that additive variation (analogous to multiplicative effects in case-control studies) will tend to dominate even when nonadditive effects exist and the impact of LD is discounted.

## THEORETICAL DERIVATIONS

### LD MODEL

Let *A* and *B* be a pair of biallelic SNPs and code the alleles at each as 0 and 1. In the situations that we examine, *A* will be a causal SNP and *B* will be a marker SNP. Let *f*_*A*_ = Pr(*A* = 1) be the frequency of allele 1 in the population at SNP *A*, and define *f*_*B*_ similarly for SNP *B*.

For brevity, we will refer to the haplotype with *A* = *i* and *B* = *j* as *ij*. Consider the population distribution of the four possible haplotypes formed by the two SNPs; three parameters are necessary to represent an arbitrary distribution. Together with *f*_*A*_ and *f*_*B*_, we use the population correlation coefficient to fully parameterize this distribution. The square of this is a commonly used measure of LD, usually denoted by *r*^2^ [e.g. Zondervan and Cardon, 2004].

Define the following conditional probabilities,


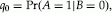
(1)


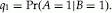
(2)

These allow the following representation of the haplotype distribution,


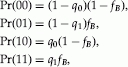


and give the identity,


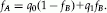


The correlation coefficient can be expressed in terms of these quantities and can be shown to be,


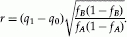


By solving these last two equations for *q*_0_ and *q*_1_, we can see that the haplotype distribution is fully and uniquely specified by *f*_*A*_, *f*_*B*_, and *r* (if they are consistent with a haplotype distribution).

As is well known, the range of *r* depends on the allele frequencies. Suppose, without loss of generality, that *f*_*A*_ and *f*_*B*_ are minor allele frequencies and that *f*_*A*_≤*f*_*B*_. By considering the possible values of *q*_0_ and *q*_1_, it can be shown that,


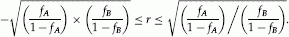
(3)

The roles of *f*_*A*_ and *f*_*B*_ swap if *f*_*A*_≥*f*_*B*_. From this we can see that in order for a high positive correlation to be possible we need to have *f*_*A*_≈*f*_*B*_, and for a high negative correlation we require *f*_*A*_ and *f*_*B*_ to both be large.^1^ A correlation in either direction will suffice for the marker to be a good surrogate. Thus, we can conclude that in situations where one of the SNPs is rare (either the marker or the causal SNP), the ability to detect associations will be impaired unless the other SNP is also rare and highly correlated.

We use the term *diplotype* to mean a pair of two-SNP haplotypes belonging to an individual. Let 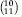 represent the diplotype comprising the two haplotypes 10 and 11 (i.e. having genotype 2 at SNP *A* and genotype 1 at SNP *B*). To obtain a diplotype distribution, we assume Hardy-Weinberg equilibrium (HWE) for haplotypes, which real data tends to follow in the context we are considering. For example, 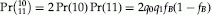. There are 10 possible diplotypes but only nine distinguishable pairs of genotypes. In particular, the genotype pair consisting of two heterozygotes can correspond to either of the two diplotypes 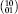 or 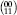. We only consider analyses using genotypes so will sum over this diplotype pair where necessary.

### DISEASE MODELS

Consider a diploid individual at a particular SNP. Let the genotype at the SNP be *G* and the disease status be *Y*, where *Y* = 1 denotes a diseased individual and *Y* = 0 denotes a healthy individual. Let *p* = Pr(*Y* = 1|*G*). Logistic regression models are commonly used to model disease risk in GWAS [e.g. Cantor et al., 2010]. The most prominent is one in which the log-odds of disease increases (or decreases) additively by β with each copy of allele 1,


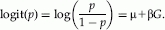


In other words, each additional copy of the risk allele increases the odds of disease by the same multiplicative factor. This is variously referred to as either the *additive* model or the *multiplicative* model. We use the latter term throughout but will refer to β as the additive parameter or effect since it naturally operates additively on the log scale. The widely used Cochran-Armitage trend test [Armitage, 1955] is the score test of the null hypothesis (β = 0) under this model [Sasieni, 1997].

The derivations we present relate disease models by comparing penetrances at marker and causal SNPs. For this purpose, it proves convenient to consider log risk regression models rather than logistic regression. For example, the multiplicative risk regression model is


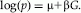


In GWAS, it is standard to use (unphenotyped) cohort or population samples in place of control samples but analyze it as a case-control study using logistic regression. This is actually equivalent to fitting a log risk regression model [Schouten et al., 1993]. Thus, log risk regression is an appropriate model to consider in this context. The two models are related analogously to the way that the odds ratio (OR) and relative risk (RR) are related, and will be approximately equivalent when the disease prevalence is relatively small.

We consider two extensions of the simple model: a general model with an extra parameter that models deviation from the simple model at the heterozygote and an interaction model with an extra parameter that models the joint multiplicative effect of the two interacting SNPs.

The general model will have three parameters and would allow a different disease risk for each genotype. Various parameterizations are possible, we use the following which is based on the multiplicative model (and is similar to that of Balding [2006]),


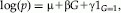


where **1**_*G* = 1_ is an indicator function that takes value 1 for heterozygotes and 0 for homozygotes. We refer to this as the *general* model. The extra parameter, γ, models the deviation from a multiplicative model at the heterozygote. We refer to it as the *dominance* parameter. Other commonly used models are special cases of this model and can be recovered by setting the dominance parameter to specific values: γ = 0 gives a multiplicative model, γ = β a dominant model, and γ = −β a recessive model (where β>0, which may be assumed without loss of generality by relabeling the alleles). To distinguish between parameters corresponding to different SNPs we label them with a subscript, e.g. β_*A*_ is the additive parameter for SNP *A*.

There are many different ways of modeling interactions [e.g. Marchini et al., 2005] and correspondingly many different parameterizations. Here we consider the simplest form from a statistical standpoint: a two-SNP model with a single additive interaction parameter,


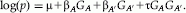


The parameter τ models deviation from the two-SNP multiplicative model and we refer to it as the *interaction* parameter.

### IMPACT OF LD ON DISEASE PARAMETERS

#### Multiplicative model

The multiplicative model is naturally defined for haplotypes as well as genotypes. Indeed, they are equivalent under the assumption of HWE [Sasieni, 1997]. For common diseases we do not expect significant deviations from HWE, and therefore turn to the haplotype setting as a simplifying device for studying genotype models. The same approach has been used by previous authors [Chapman et al., 2003; Pritchard and Przeworski, 2001; Zondervan and Cardon, 2004].

Let SNP *A* be causal and SNP *B* be a marker. Define the following disease penetrances:


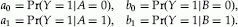


We can relate the penetrances at the two SNPs by using the LD model. In particular, using Equations (1) and (2),


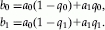


Taking the difference gives a convenient summary of the relationship,


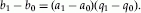


Re-writing this in terms of the disease model parameters, allele frequencies and LD gives,


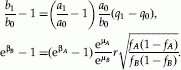


We can derive a simpler expression when effect sizes are small. Using the approximation e*^x^*−1≈*x*, and also µ_*A*_≈µ_*B*_ (which is equivalent to saying the penetrances at allele 0 are similar at the two SNPs), we have,


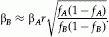


We see that the additive effect at the marker SNP decreases linearly with *r* as the LD becomes weaker. This is a key result: it gives an intuitive and convenient relationship between the parameters of interest. Furthermore, the relationship later derived for the effect of LD on power follows directly from it. In this formulation, this result appears to be novel.

Zondervan and Cardon [2004] derive a similar formula, but expressed in terms of different parameters. They parameterize LD in terms of the disequilibrium coefficient, *D* = Pr(11) − *f_A_f_B_*, instead of *r*, and use the OR instead of the RR (recall that we are using a log risk regression model),


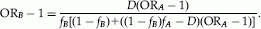


#### General model

Let *A* and *B* now represent genotypes (note that the haplotype approximation and corresponding HWE assumption we used above are thus not required). Define the following disease penetrances:


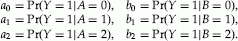


As before, relating the penetrances using the LD model gives,


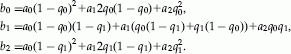


The expression 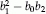 is a measure of the deviation from a multiplicative model (for which it is exactly 0), and has a simple form that relates the marker and causal SNP penetrances,


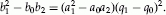


Re-writing this in terms of the disease model parameters, allele frequencies and LD gives,


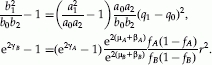


When the dominance effect is small, we can derive a simpler expression using the approximations e*^x^*−1≈*x* and μ_*A*_ + β_*A*_ ≈ μ_*B*_ + β_*B*_,


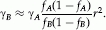
(5)

We see that the dominance effect at the marker SNP decreases quadratically with *r* as the LD becomes weaker. Analogous to Equation (4), this is a key result and in this formulation it appears to be novel. Sham et al. [2000] derive a similar result relating variance components in models of quantitative traits; our derivation here relates parameters in models of case-control data. The formula gives an intuitive and convenient relationship between the parameters of interest, and the relationship later derived for the effect of LD on power follows directly from it. Crucially, this result contrasts with that for the additive parameter, with the dependence on LD being through *r*^2^ rather than *r*.

GWAS analyses typically employ the trend test, which effectively fits a multiplicative model. While this may result in model mis-specification (if the model underlying the data is not multiplicative), it will nevertheless pick up some of the association signal. For a given underlying disease model, allele frequency, and ratio of cases to controls in the sample, there will be a characteristic mean value for the additive parameter when fitting the multiplicative model. We refer to this as the *effective* additive parameter and denote it by β′. It can be calculated numerically by fitting the multiplicative model, using logistic regression, to the theoretical genotype frequencies for cases and controls under the disease model of interest, weighted by the case-control sampling ratio. In other words, we pretend the theoretical frequencies are sample counts. To see why this works, imagine taking a very large case-control sample: the resulting estimate of β′ will be very close to its mean, and the genotype counts will closely match the underlying genotype frequency distribution. In the logistic regression fit, point estimates only depend on relative frequencies of the different genotype/phenotype classes (although estimates of uncertainty will also depend on the absolute counts). Specifically, increasing the counts but keeping the relative ratios the same is equivalent to scaling the log-likelihood by a constant—it will make it more peaked but not change the location of the mode.

**Fig. 1:**
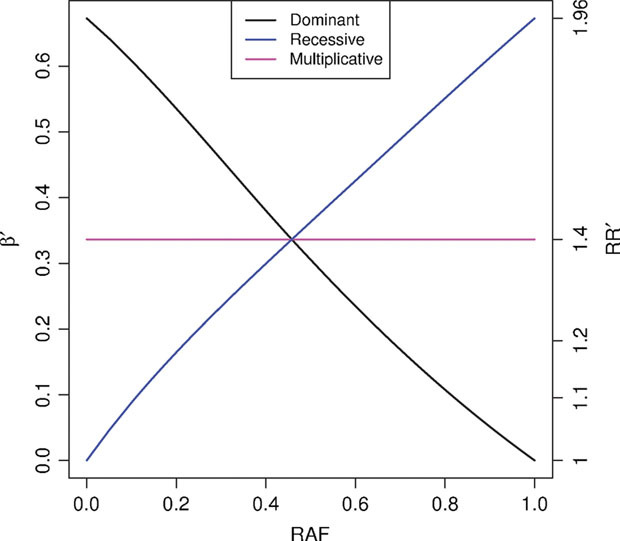
The effective additive parameter for three disease models, plotted against the RAF. A homozygous RR of 1.4^2^ and an equal number of cases and controls were assumed for all disease models. The right-hand *y*-axis shows the per-allele RR corresponding to each value of β′ (i.e. RR′ = e^β^′). Note that for the multiplicative model, β′ = β = log(1.4) for all RAFs. RAF, risk allele frequency; RR, relative risk.

Figure 1 shows how the effective additive parameter for a few models varies depending on the allele frequency. Here we have assumed an equal number of cases and controls in the sample; varying this ratio gives qualitatively similar results and is therefore a less important factor than the allele frequency (data not shown). One way to understand the results is think of them as similar to a weighted average of the disease risks at each genotype. When the allele frequency is at one extreme, only two of the three possible genotypes will be represented in the sample, and the model fit will be based mainly on the difference in risk between these two. Thus, for both the dominant and recessive models the limiting values are either zero effect, when the two equal-risk genotypes predominate, or a large effect, when the two genotypes differ in risk. In the later case, the effect is double (on the log scale) that of the multiplicative model which has the same homozygous RR as the original dominant/recessive model.

Note that the diagonal lines in this plot are actually not symmetric—they intersect at a risk allele frequency less than 0.5, and reflections neither vertically nor horizontally will make them match. We may have assumed that there should be symmetry, for example by interchanging the cases and controls to switch between dominant and recessive model. However, this is not valid since they are ascertained differently, the controls being a sample from the whole population and the cases from the diseased subset.

**Fig. 2:**
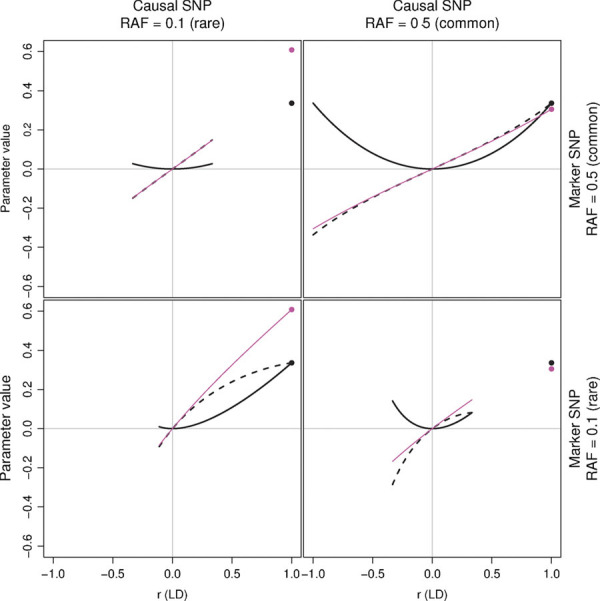
Impact of LD on disease model parameters for a dominant model. Parameter values as functions of *r*, for a selection of RAFs. A dominant model with a homozygous RR of 1.4^2^ at the causal SNP is assumed, corresponding to general model parameter values of β_*A*_ = γ_*A*_ = log(1.4) = 0.34. The solid black line shows the dominance parameter (γ_*B*_), the dashed black line the additive parameter (β_*B*_), and the magenta line the effective additive parameter (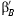) at the marker SNP. The respective parameter values at the causal SNP are shown by points at *r* = 1, following the same color scheme as the lines (in this case, the points for β_*A*_ and γ_*A*_ overlap since they have the same value). Plots in each row correspond to a given marker SNP RAF and columns to a given causal SNP RAF, as labeled. The range of possible values of *r* depends on the allele frequencies, as shown by Equation (3). Note that a negative value for β is equivalent to a positive value for it when considered with respect to the other allele at the SNP. RAF, risk allele frequency; LD, linkage disequilibrium; RR, relative risk; SNP, single nucleotide polymorphism.

**Fig. 3:**
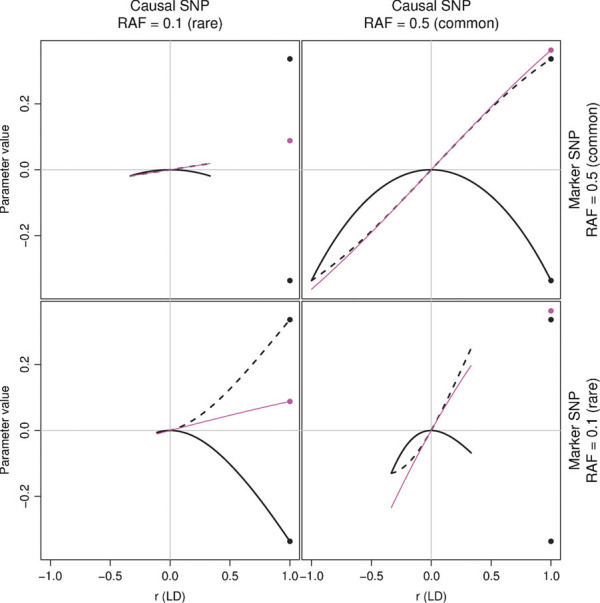
Impact of LD on disease model parameters for a recessive model. Same as Figure 2, but now for a recessive model with a homozygous RR of 1.4^2^, corresponding to general model parameter values of β_*A*_ = −γ_*A*_ = log(1.4) = 0.34. LD, linkage disequilibrium; RR, relative risk.

Figures 2 and 3 show the effect of LD and allele frequency on the disease model parameters, for dominant and recessive models, respectively. The parameter values at the marker SNP were calculated using,


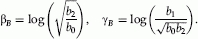
(6)

The figures also show the effective additive parameters, 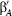 and 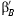, calculated using logistic regression as described above. Thus, in these figures we have plotted the exact values for all parameters, rather than approximations based on Equations (4) and (5). We can see that the approximations accurately describe the observed behavior, with the dominance effect decaying faster than the additive effects, approximately quadratically vs. linearly.

**Fig. 4:**
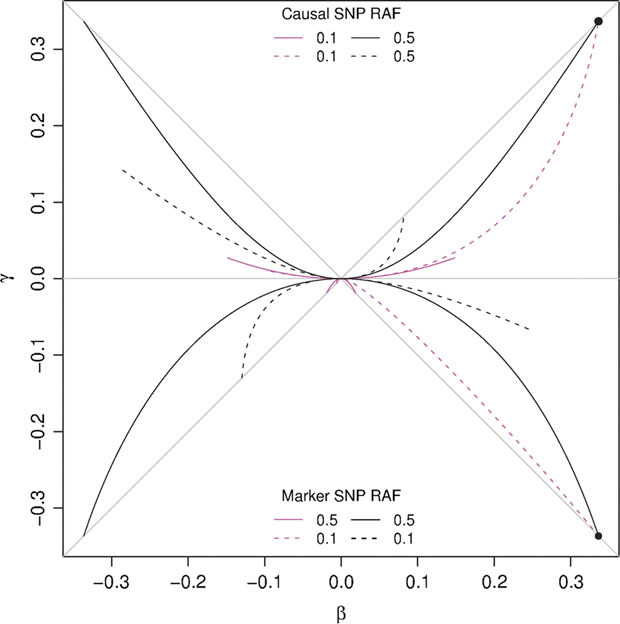
Model space plot showing distortion toward a multiplicative model. The two disease parameters (dominance vs. additive; γ vs. β) plotted against each other showing the full space of models up to the value of the baseline parameter (µ). The horizontal gray line shows the subspace of multiplicative models. The gray lines above the horizontal show the subspace of dominant models, and those below show the subspace of recessive models. Curves and points trace out the models for the scenarios shown in Figures 2 and 3, lying above and below the horizontal line, respectively. Curves are drawn in different styles to show the causal and marker SNP RAFs they correspond to, as shown by the two legends. The two points represent the true disease models at the causal SNP. SNP, single nucleotide polymorphism; RAF, risk allele frequency.

Another and perhaps more natural way to see the effect of LD is to plot the two disease parameters against each other. We refer to this as a *model space* plot, since each point corresponds to a particular disease model and all possible models can be represented in this way (up to the value of µ). Figure 4 shows such a plot with curves for each of the eight scenarios shown in Figures 2 and 3. The subspace of multiplicative models is shown by the horizontal line, and the null model is at the origin. The curves trace out the theoretical disease model at the marker SNP, with lower LD corresponding to points closer to the origin along these curves. We can now clearly see how LD acts to make the observed model more multiplicative—notice that the curves “bend” toward the horizontal line.

#### Interaction model

Like the multiplicative model, the interaction model we use is naturally defined for haplotypes as well as genotypes and we again turn to the haplotype setting as a simplifying device. Let SNPs *A* and *A*′ be causal and SNPs *B* and *B*′ be their tag SNPs, respectively. Define the following disease penetrances:


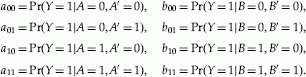


Let 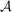 and 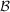 denote the 2×2 matrices of penetrances with entries as above, and 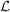 and 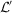 denote the following matrices of LD parameters,


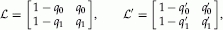


where the former describe the LD between SNPs *A* and *B* and the latter the LD between SNPs *A*′ and *B*′. Using the LD model,


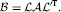


The determinant, 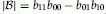, is exactly 0 for a two-SNP multiplicative model and is a convenient measure for the deviation from it. Since 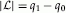 and 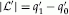, we obtain,


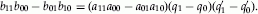


We can re-write this in terms of the disease model parameters, allele frequencies, and LD,


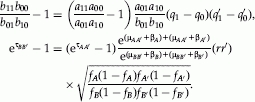


When the interaction effect is small, we can derive a simpler expression using the approximations e*^x^*−1≈*x*, and 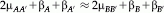,


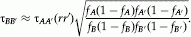
(7)

The interaction effect at the marker SNPs decreases quadratically with LD, analogous to the dominance effect. The quadratic factor is a product of the correlation due to each of the tag SNPs. This is again a key result, showing how a simple type of statistical interaction decays with multiple sources of LD, and the relationship later derived for the power to detect the interaction follows directly from it. Crucially, this result contrasts with that for the additive parameter, the decay with LD being quadratic rather than linear.

### IMPACT OF LD ON POWER

The previous section describes the impact of LD on the disease effect parameters. We now examine how this impacts the power of the corresponding tests. Derivations of the noncentrality parameters for each test are shown in Appendix A. Combining these with the parameter-LD relationships from the previous section allows us to give approximate expressions for the power when testing at marker SNPs.

#### Trend test

Suppose we have a case-control sample of size *N*_*A*_ that types the causal SNP and also one of size *N*_*B*_ that types a marker SNP. From Equation (10), a trend test at the causal SNP has noncentrality parameter,


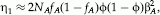


where φ is the proportion of cases in the sample. Applying Equation (4), the same test at the marker SNP has noncentrality parameter,


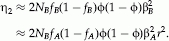


Comparing η_1_ and η_2_, we see that a sample size of *N*_*B*_ = *N*_*A*_/*r*^2^ is required to achieve the same power as typing the causal SNP directly. This is essentially the same derivation as shown in Pritchard and Przeworski [2001], but here based on the Wald test.

#### Deviation test

The Wald test for the dominance parameter amounts to comparing the multiplicative and general models and thus tests for a deviation from the multiplicative model. We therefore refer to this as the *deviation* test. Applying the same idea as above, now using Equations (11) and (5), gives the noncentrality parameters,


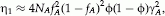


and


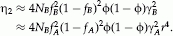


Thus, a sample size of *N*_*B*_ = *N*_*A*_/*r*^4^ is required to achieve the same power as typing the causal SNP directly.

#### Interaction test

The Wald test for the interaction parameter compares our interaction model to a two-SNP multiplicative model; we refer to this as the *interaction* test. Using Equations (12) and (7) gives the noncentrality parameters,


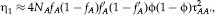


and


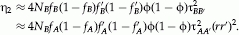


Thus, a sample size of *N*_*B*_ = *N*_*A*_/(*rr*′)^2^ is required to achieve the same power as typing the causal SNPs directly.

## SIMULATION STUDY

Due to the complex LD structure in the human genome, and also ascertainment effects from GWAS study designs, it is difficult to evaluate the impact of distortion on GWAS results analytically. For this reason, we also adopted a simulation approach, using existing data and methods to simulate realistic GWAS samples under various disease models.

### METHOD

We took data from the 10 ENCODE regions [ENCODE Project Consortium, 2004] within the Caucasian (CEU) analysis panel of HapMap II [International HapMap Consortium, 2007], which have undergone SNP ascertainment by resequencing. These regions therefore show a fuller spectrum of SNPs than are represented in the HapMap data at large, and haplotypes are expected to be accurate due to the trio design of the HapMap panels [International HapMap Consortium, 2005]. We used the HAPGEN software package [Spencer et al., 2009] to produce a population of 100,000 haplotypes based on the empirical LD patterns in HapMap II. This haplotype panel served as the base for our GWAS simulations.

For a given disease model of interest, each allele at each SNP in each ENCODE region was in turn presumed causal, and a complete association and replication study for each (20,968 in total) was simulated according to the following procedure.

We generated a sample of 2,000 diploid cases and 2,000 diploid controls from the panel as follows. For the controls, we sampled haplotypes uniformly from the panel (without replacement) and combined them in pairs. For the cases, we sampled haplotypes according to the genotype frequencies at the causal SNP as dictated by the disease model. Specifically, we first simulated genotypes at the causal SNP by sampling with probabilities proportional to:


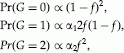


where α_1_ and α_2_ are, respectively, the RRs of genotypes 1 and 2 relative to genotype 0, and *f* is the frequency of allele 1 in the panel. We then sampled pairs of haplotypes (without replacement) uniformly from the panel such that they were consistent with the genotypes.

The next step was to thin the SNPs down to a set that would be present on a typical genotyping chip; we used the Affymetrix Genome-wide Human SNP Array 6.0 (Affymetrix, Santa Clara, CA) for this purpose. Examining only SNPs on this chip, for each we applied the trend test and calculated a *P*-value. We then took SNP with the smallest *P*-value, which we refer to as the *hit* SNP, and checked whether it showed a *P*-value less than 1×10^−6^. If this occurred we then modeled a replication study at this SNP using an additional 2,000 cases and 2,000 controls, and required a *P*-value less than 0.01. In what follows we only considered those simulations where the hit SNP on the genotyping chip met both criteria, as these model the ascertainment implicit in reported GWAS associations.

The final step was to evaluate the impact in terms of distortion. For each simulation run where an association was detected, we applied the deviation test to the hit SNP using the genotype counts from the replication scan and checked if a *P*-value less than 0.05 was obtained. This procedure is typical of what is applied in GWAS [e.g. Wellcome Trust Case Control Consortium, 2007]. Thus, there are three possible overall outcomes from each simulation: (i) no association detected; (ii) association detected but not deviation; and (iii) both association and deviation detected.

Effect sizes were estimated by maximum likelihood using the R statistical software package [R Development Core Team, 2009].

We ran simulations for a range of RRs, using multiplicative, recessive, and dominant disease models. While there are many possible disease models we might consider, these represent extreme ends on the scale of deviations that we would generally expect to observe in real studies.

For simplicity, we only ran simulations with single-SNP disease models. Since we showed theoretically that dominance and interaction effects have the same order decay, we expect that simulations with interaction effects to show similar results to what we learn about dominance effects here.

### RESULTS

**Fig. 5:**
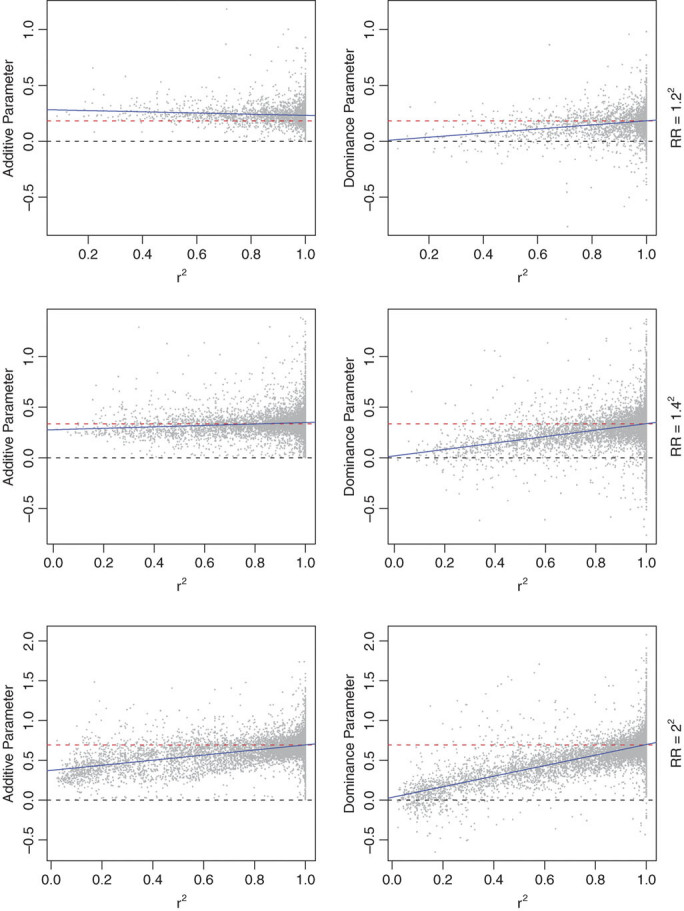
Parameter estimates and LD from simulations for a dominant model. Estimates of the additive and dominance parameters respectively (by column) at the hit SNP, plotted against the *r*^2^ between the causal and hit SNPs. The estimates are from the simulated replication sample from simulations with a dominant causal SNP with homozygous RRs of 1.2^2^, 1.4^2^, and 2^2^, respectively (by row; corresponding to true parameter values of β = γ = 0.5 log(Hom. RR) = 0.18, 0.34, 0.69). Only simulations where the hit SNP passed the scan and replication criteria are displayed. The dashed red lines denote the true parameter values. The dashed black lines indicate a zero effect. The blue lines show linear regression fits to the points on each plot, to aid visual comparisons. LD, linkage disequilibrium; SNP, single nucleotide polymorphism; RR, relative risk.

Figure 5 shows how the additive and dominance parameter estimates at the hit SNPs vary with LD, for simulations where the causal SNP is dominant. As predicted by theory, the dominance parameter tends toward the null value of 0 at a faster rate than does the additive parameter. Note that these plots show data covering the entire range of causal allele frequencies in the ENCODE regions, unlike the theoretical curves (Figs. 2–4), which are only for two specific values.

**Fig. 6:**
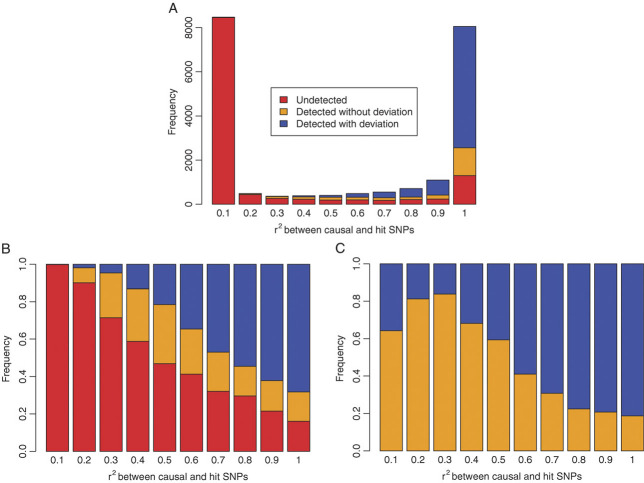
A breakdown of the simulation results by outcome and LD, for simulations with a dominant model with a homozygous RR of 1.4^2^. The LD is shown as the *r*^2^ between the causal and hit SNPs, split into bins of width 0.1 (labeled on the *x*-axis with the highest possible *r*^2^ value for each bin). The three possible outcomes are: the hit SNP does not pass the scan and replication criteria (“Undetected”); that it passes these criteria but a subsequent deviation test is not significant (“Detected without deviation”); or that this test is significant (“Detected with deviation”). For each LD bin: panel **A** shows the absolute counts of each outcome, panel **B** shows their relative proportions, while panel **C** shows the relative proportions of the last two outcomes only. Note that the two leftmost columns in panel C are based on very small counts and so the exact values plotted are not precise estimates of the relative proportions. LD, linkage disequilibrium; SNP, single nucleotide polymorphism; RR, relative risk.

Table I shows the distribution of the three outcomes for simulations across different disease models and RRs. We see that much of the time when we detect association, the deviation test will also give the correct outcome, even at the smaller effect sizes. This is despite the distortion effect observed above. The reason for this is that the LD between the causal and hit SNPs is often quite high, and thus will not suffer from much distortion. Figure 6A shows a typical LD distribution for a set of simulations—most of the time the hit SNP is at the extremes of the LD spectrum. Correspondingly, Figure 6B shows the distribution of outcomes for a given amount of LD, and Figure 6C shows the outcome of the deviation test among detected associations only. We see that, as the LD decreases, the relative amount of distortion among detected associations gradually increases. The overall proportion of associations detected without deviation may seem slightly small (i.e. the yellow bars in Fig. 6B), but note that this is in a sense “competing” with the no-association outcome as the LD decreases, so will only represent the small window of outcomes where γ is diminished sufficiently to make it hard to detect but where β′ is not.

**Table I:** Power estimates from simulations

		Outcome (%)			
			
Model	Hom. RR	Undetected	Assoc. only	Assoc. + deviation	Deviation detection rate among associations (%)
Multiplicative	1.1^2^	100	0	0	–
Multiplicative	1.2^2^	94	5	0	5
Multiplicative	1.3^2^	70	29	2	6
Multiplicative	1.4^2^	49	49	2	5
Multiplicative	1.5^2^	39	59	3	5
Multiplicative	2.0^2^	23	73	4	5
Dominant	1.1^2^	100	0	0	–
Dominant	1.2^2^	84	9	7	46
Dominant	1.3^2^	64	12	24	68
Dominant	1.4^2^	56	10	34	77
Dominant	1.5^2^	51	10	39	80
Dominant	2.0^2^	41	9	51	86
Recessive	1.1^2^	100	0	0	–
Recessive	1.2^2^	84	8	7	47
Recessive	1.3^2^	62	12	26	69
Recessive	1.4^2^	52	11	37	76
Recessive	1.5^2^	46	11	43	79
Recessive	2.0^2^	32	11	58	85

The distribution of simulation outcomes over a range of disease models and effect sizes. The effect size is given by the homozygous RR (“Hom. RR”), which compares the risk of the two homozygotes. Each row shows results aggregated across the 20,968 simulations for a given disease model and effect size, effectively averaging over the allele frequency distribution in the ENCODE regions. The three possible outcomes from each simulation are: the hit SNP does not pass the scan and replication criteria (“Undetected”); that it passes these criteria but a subsequent deviation test is not significant (“Assoc. only”); or that this test is significant (“Assoc. + deviation”). The final column shows the proportion of simulations for which deviation was detected among those for which an association was detected (omitted for the smallest effect size due to very small numbers of detected associations). All figures are rounded to the nearest percentage. RR, relative risk; SNP, single nucleotide polymorphism.

The use of the trend test induces an ascertainment bias in favor of additive effects. A natural alternative is to use the test with 2 degrees of freedom that compares the general model with the null model, which we refer to as the *general* test. There are merits, but also disadvantages, to using this test (see Discussion). Since GWAS are typically analyzed with the trend test, here we focused only on results from simulations based on that test.

The results we have shown here are for a given sample size and range of effect sizes. Since power depends on both of these factors in a simple way, they are also more generally applicable. Specifically, the noncentrality parameter is proportional to *N*θ^2^, where θ is the parameter of interest (see Equations (A.3)–(A.5) in Appendix A). For example, if one is interested in what happens for a sample size of 2*N*, then the same qualitative results would be obtained for 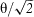 as were obtained for θ with sample size *N*. Thus, it is sufficient to conduct simulations for only one sample size to yield conclusions that hold more generally.

## DISCUSSION

The correlation along the human genome has allowed GWAS to look for regions associated with disease without having to genotype with all known genetic variants. Although this approach has been successful, it entails that observed GWAS associations will often only be surrogates for the casual variants and will typically represent a noisy measurement of them. One consequence of this is that the disease model as inferred from associated loci may be a distorted version of the true disease model. Through analytical derivations, we have characterized the relationship between disease model parameters and LD, and the resulting impact on power. These show that dominance interaction effects tend to decay quickly, and that such distortions therefore tend to make the disease model look more like a multiplicative model as the correlation between causal and hit SNPs decreases.

To quantify the effect of distortion on observed GWAS outcomes, we ran an extensive simulation study designed to mimic patterns of LD in European Caucasian populations. We considered recessive and dominant models, both representing natural extremes for deviation away from a multiplicative model. We were specifically interested in the power of detecting such deviations, and also ran simulations under the multiplicative model for comparison.

Our analyses showed that if the true model is recessive or dominant, but the locus is nonetheless detected by using the trend test, then a standard test will often also successfully detect deviation from a multiplicative model. Informally, for the relatively small effect sizes typical at GWAS loci, the effect is unlikely to be detected unless the causal variant is relatively common and well tagged by the SNPs on the chip. The high correlation between the causal and hit SNPs then means that there is reasonable power to detect deviation from the multiplicative model, even under model distortion. While encouraging, we note, first, that the dominant and recessive models are extreme, and power to detect nonmultiplicative models, which are “closer” to the multiplicative model, will be lower. Second, as our simulations show, there will be settings where the model distortion is such that under the recessive and dominant models the locus is not detected at all using the trend test.

Nearly 3,000 disease associations from GWAS have been published in the past few years [Hindorff et al., 2010]. Relatively few of these are known to follow specific, nonmultiplicative models. It may be that testing for deviations is not done routinely, although even in studies where such investigations have been carried out, few SNPs have shown convincing evidence of recessive or dominant effects [e.g. Wellcome Trust Case Control Consortium, 2007]. Our simulations have shown that such effects will often be detectable, and therefore it is worth explicitly testing associated loci for deviations. As noted above, real disease effects may not deviate as much as fully recessive and dominant effects, and small deviations from multiplicativity will be relatively hard to detect, and easily disguised with only a slight amount of distortion.

One consideration in the analyses of GWAS data is which statistical test or model to use for the initial genome-wide scan. Since we expect to detect SNPs that are affected to a greater or lesser extent by distortion, a sensible default choice is the trend test, which is well-powered for multiplicative effects. It also has the benefit of being more robust to genotyping error than, for example, the general two degree of freedom test [Ahn et al., 2007]. We note that others have also made similar recommendations [Cantor et al., 2010; Iles, 2008]. Nevertheless, the trend test can be usefully complemented by the general test [Wellcome Trust Case Control Consortium, 2007], or other approaches for investigating nonmultiplicative models, such as the deviation test. The corresponding advice for Bayesian analyses is to place most of the prior weight on multiplicative models, and spread the rest out more widely [Stephens and Balding, 2009].

## Footnotes

1 This (apparent) asymmetry is due to the choice of *f*_*A*_ and *f*_*B*_ as being the *minor* allele frequencies.

## APPENDIX A: DERIVATION OF THE NONCENTRALITY PARAMETERS

We derive noncentrality parameters for the three testing scenarios outlined in the main text: the trend test, the deviation test, and the interaction test. In each case, we consider a Wald test using a logistic regression model. While a score test is standard in the first scenario, deriving the noncentrality parameter for such a test gives the same result (derivation not shown), and the two tests are nonetheless asymptotically equivalent [Cox and Hinkley, 1974].

Let the number of cases and controls be *S* and *R*, respectively, the total number of individuals in the sample be *N*, and the proportion of cases in the sample be φ = *S*/*N*. At a given SNP, let the number of individuals with genotypes 0, 1, and 2 be *n*_0_, *n*_1_, and *n*_2_, respectively. Let the subscript *i* refer to individual *i*.

### TREND TEST

For convenience, we reparameterize the multiplicative model by mean-centering the genotypes,

where  is the genotype mean and  is the new baseline parameter. This makes the parameterization “null orthogonal” as defined by Kass and Vaidyanathan [1992], who show that, in what follows, we may assume that the Fisher information matrix is approximately diagonal. Note that the β parameter is unchanged (it only depends on the *differences* between genotypes between individuals and these are unchanged after mean-centering), but µ has been replaced by ν. We denote the mean-centered genotypes by . Note that .

The likelihood function is . Let *l* = log *L* be the log-likelihood. Since the Fisher information matrix is approximately diagonal, the likelihood approximately factorizes into components attributable to each parameter. Thus, we only need to consider the submatrix corresponding to *β*, which can be shown to be,

(A.1)

We now propose further approximations to this expression. First, we approximate the logistic function by a Taylor expansion about ν and apply it to the regression probabilities,

Under the null, by design we have,

This will be a good approximation under the alternative as well—it can be shown that the MLE of ν satisfies this equation up to terms . This gives a simpler expression for the Taylor expansion,

A useful expression derived from this is,

(A.2)

Note that the terms containing (1−2φ) disappear when φ = 1/2 (equal number of cases and controls), meaning that these approximations are particularly good in that case—e.g.  becomes  in the last equation.

Applying the expansion from Equation (9) to Equation (8) gives,

The reciprocal of this is the asymptotic variance of the MLE of β, . The Wald test statistic for β asymptotically follows a  distribution with noncentrality parameter . Therefore,

When effect sizes are small, as is the norm for GWAS, the  terms become negligible and may be omitted. We can also further simplify this expression by assuming HWE and taking the expectation over the genotypes,

which gives,

(A.3)

Chapman et al. [2003] derive a similar result, with their formula expressed in terms of allele frequencies in cases and controls rather than the disease effect parameters directly.

### DEVIATION TEST

Considering the general model, we follow an analogous derivation to the above. The mean-centered reparameterization is,

with *n*_1_/*N* being the mean of **1**_*G*_*i*_ = 1_ across the sample, and . Let  and *B_i_* = **1**_*G*_*i*_ = 1_ − *n*_1_/*N*. Note that . With this parameterization, we only need to consider the Fisher information submatrix corresponding to the disease effect parameters, β and γ,

A two-dimensional Taylor expansion similar to Equation (9) gives,

and lets us simplify the Fisher information,

Assuming HWE we have,

Replacing the terms in the matrix above with these expectations gives,

Inverting and taking the bottom-right element gives the asymptotic variance of ,

The Wald test statistic for γ asymptotically follows a  distribution with noncentrality parameter . Therefore,

When effect sizes are small, as is the norm for GWAS, the  terms become negligible and may be omitted,

(A.4)

### INTERACTION TEST

We follow an analogous derivation to the above using the interaction model. We will assume that the genotypes at the two SNPs in the model are independent (i.e. in linkage equilibrium). This is the simplest scenario and will generally hold for SNPs that are distant to each other or on separate chromosomes. When there is LD between the two SNPs, the ability to observe interaction is impaired because some of the genotype combinations become less frequent. In the extreme scenario of complete LD, interaction cannot be observed at all.

For notational convenience, we denote the genotypes at the two SNPs by *G* and *H*, respectively, and the additive parameters by β_1_ and β_2_, respectively. The mean-centered reparameterization is,

with *M*_*i*_ = *G*_*i*_*H*_*i*_ and  being its mean across the sample. Let , and *C*_*i*_ = *G*_*i*_*H*_*i*_−*M*. Note that . With this parameterization, we only need to consider the Fisher information submatrix corresponding to the disease effect parameters, β_1_, β_2_, and τ,

A three-dimensional Taylor expansion similar to Equation (9) gives,

and lets us simplify the Fisher information,

Assuming HWE, we have

where *f* and *f*′ are the allele frequencies of the two SNPs. These expectations are derived as previously, based on the variances and covariances of the quantities *G*, *H*, and *M*. Replacing the terms in the matrix above with these expectations gives,

Inverting and taking the bottom-right element gives the asymptotic variance of ,

The Wald test statistic for τ asymptotically follows a  distribution with noncentrality parameter . Therefore,

When effect sizes are small, as is the norm for GWAS, the  terms become negligible and may be omitted,

(A.5)
